# Facilitating systems-level analyses of all-cause and Covid-mediated sepsis through SeptiSearch, a manually-curated compendium of dysregulated gene sets

**DOI:** 10.3389/fimmu.2023.1135859

**Published:** 2023-05-26

**Authors:** Arjun S. Baghela, Jasmine Tam, Travis M. Blimkie, Bhavjinder K. Dhillon, Robert E.W. Hancock

**Affiliations:** Centre for Microbial Diseases and Immunity Research, University of British Colombia, Vancouver, Canada

**Keywords:** database, gene set analysis, transcriptomics, inflammation, sepsis, systems immunology

## Abstract

**Background:**

Sepsis is a dysfunctional host response to infection. The syndrome leads to millions of deaths annually (19.7% of all deaths in 2017) and is the cause of most deaths from severe Covid infections. High throughput sequencing or ‘omics’ experiments in molecular and clinical sepsis research have been widely utilized to identify new diagnostics and therapies. Transcriptomics, quantifying gene expression, has dominated these studies, due to the efficiency of measuring gene expression in tissues and the technical accuracy of technologies like RNA-Seq.

**Objective:**

Most of these studies seek to uncover novel mechanistic insights into sepsis pathogenesis and diagnostic gene signatures by identifying genes differentially expressed between two or more relevant conditions. However, little effort has been made, to date, to aggregate this knowledge from such studies. In this study we sought to build a compendium of previously described gene sets that combines knowledge gained from sepsis-associated studies. This would enable the identification of genes most associated with sepsis pathogenesis, and the description of the molecular pathways commonly associated with sepsis.

**Methods:**

PubMed was searched for studies using transcriptomics to characterize acute infection/sepsis and severe sepsis (i.e., sepsis combined with organ failure). Several studies were identified that used transcriptomics to identify differentially expressed (DE) genes, predictive/prognostic signatures, and underlying molecular responses and pathways. The molecules included in each gene set were collected, in addition to the relevant study metadata (e.g., patient groups used for comparison, sample collection time point, tissue type, etc.).

**Results:**

After performing extensive literature curation of 74 sepsis-related publications involving transcriptomics, 103 unique gene sets (comprising 20,899 unique genes) from thousands of patients were collated together with associated metadata. Frequently described genes included in gene sets as well as the molecular mechanisms they were involved in were identified. These mechanisms included neutrophil degranulation, generation of second messenger molecules, IL-4 and -13 signaling, and IL-10 signaling among many others. The database, which we named SeptiSearch, is made available in a web application created using the Shiny framework in R, (available at https://septisearch.ca).

**Conclusions:**

SeptiSearch provides members of the sepsis community the bioinformatic tools needed to leverage and explore the gene sets contained in the database. This will allow the gene sets to be further scrutinized and analyzed for their enrichment in user-submitted gene expression data and used for validation of in-house gene sets/signatures.

## Introduction

Sepsis is recognized as a complex syndrome and defined as the dysfunctional host response to infection. It has been found to afflict 49 million people annually with 11 million deaths (19.7% of all deaths in 2017) and is the cause of most deaths from severe Covid infections (1, 2). In order to identify new opportunities for diagnostics and treatment, there has been a notable rise in the use of high-throughput omics assays to characterize patients with all-cause sepsis and in more recent years, Covid-19-associated sepsis (3). This has led to increased knowledge of the molecules (e.g., genes, proteins, metabolites) implicated in sepsis pathogenesis and patient severity. Interestingly, however, this has not resulted in a particular consensus in the literature regarding the underlying mechanisms of sepsis, an observation that we have proposed is due to the massive heterogeneity of sepsis (4), coupled with factors such as the selection of differing subsets of patients and sampling at different stages of disease and in patients of different age categories (pediatric/adult/senior). The vast majority of studies using omics to characterize sepsis patients (or *in vitro* models mimicking sepsis conditions) have performed transcriptomics (usually RNA-Seq or microarrays) to quantify gene expression. However, there has also been a rise in the use of metabolomics and proteomics (5). Typically, two or more relevant patient groups or conditions are collected (e.g., sepsis vs. non-sepsis, organ dysfunction vs. no organ dysfunction, etc.) at various stages of disease (emergency room, ICU entry, onset of septic shock etc.), and their gene expression profiles are compared to various controls (e.g. non-septic patients with sudden inflammatory response syndrome [SIRS], non-infected individuals, surgical controls or healthy volunteers, etc.) to identify differentially expressed (DE) genes. These DE genes are then characterized using functional enrichment analyses primarily using pathway (e.g., Reactome, Kyoto Encyclopedia of Genes and Genomes [KEGG], Molecular Signatures Database [MSigDB]) or Gene Ontology [GO] databases.

Such studies have also been used to identify or propose novel diagnostic biomarkers and multi-molecule signatures in an attempt to guide the clinical management of sepsis patients. Signatures are usually composed of tens to hundreds of differentially expressed genes and are obtained through various statistical and machine learning methods. In the context of sepsis, such signatures have been proposed to predict severe sepsis and/or risk stratify, identify mechanistically distinct subgroups (i.e., endotypes), and guide therapeutic interventions, among other uses (3, 6). For example, our group recently described the identification of gene expression signatures predictive of sepsis severity, including those reflecting endotypes and cross-cutting severe sepsis and mortality signatures, in global cohorts of hospital patients profiled using RNA-Seq (4). Intriguingly, across the many omics publications on sepsis, a wide variety of quite different signatures have been obtained.

Here we describe the establishment of the database SeptiSearch (www.septisearch.ca), developed to aggregate knowledge from previously-published gene expression publications, including DE genes and signatures derived therefrom, enabling rapid comparisons of these different gene sets. The SeptiSearch database was created to (1): aggregate gene sets (i.e., DE genes and signatures) associated with sepsis pathogenesis or severity, enabling users to examine sets of genes commonly associated with studies involving various experimental metadata and patient characteristics (e.g. tissue, age group, and timepoint of analysis, infection type including Covid-19 infection, etc.) (2); identify over-represented pathways among the gene sets; and (3) permit their analysis in the context of user-collected gene expression data for enrichment analysis. The current version of SeptiSearch is the first database of its kind, containing 103 curated gene sets that have biological relevance and/or predictive utility in sepsis. We found that genes commonly included in these gene sets were associated with several biological pathways, notably, Neutrophil degranulation and several interleukin and T-cell signaling pathways. Thus these mechanisms are consistently dysregulated across transcriptomic studies, strengthening their link to sepsis pathogenesis. We also present a use-case of the database, where we analyzed the gene sets in a previously-published study of an in-house cohort of sepsis patients (4), to determine if the curated gene sets were collectively expressed, and whether the genes in the signatures we described were found in previous studies. The gene sets have been made available in an R Shiny-based web application at www.septisearch.ca in order to support their dissemination to the broader sepsis community. Furthermore, these gene sets can be analyzed and manipulated within the SeptiSearch database for further analysis using gene set enrichment statistics and visualization tools.

## Methods

### Literature curation of sepsis-associated gene sets

There has been a substantial increase in the number of transcriptomics studies identifying dysregulated gene sets and signatures in sepsis patients. We sought to build a database of previously-described gene sets that combined knowledge gained from sepsis-associated studies for use by the sepsis research and informatics community. We reasoned that literature curation offers a feasible means to access gene sets rather than performing a logistically complicated meta-analysis of each author’s raw gene expression data.

From 2020-2022, PubMed was searched for studies using transcriptomics to characterize acute infection/sepsis and severe sepsis (i.e., sepsis combined with organ failure). In PubMed, the following search terms were used to identify studies of interest: (“sepsis” OR “septic shock” OR “severe sepsis”) AND (“transcriptomics” OR “gene expression profiling” OR “microarray” OR “RNA-Seq”) AND (“biomarker” OR “signature” OR “differential expression” OR “gene set”). There were 184 studies identified that described the use of transcriptomics to identify differentially expressed (DE) genes and examine underlying molecular responses, pathways and/or predictive/prognostic signatures. Most of these studies involved comparing patients with sepsis and those with sterile inflammation (i.e., Systemic Inflammatory Response Syndrome [SIRS] without evidence or suspicion of infection) or comparing sepsis to healthy control patient groups, although other comparators were utilized. These comparisons were used to identify DE gene sets and the majority (particularly older studies) employed microarray gene expression profiling which is a less comprehensive and accurate methodology than RNA-Seq (7, 8). Clearly the technique used would impact on the number and accuracy of DE genes associated with sepsis. The specific molecules included in gene sets were collected, in addition to the relevant metadata, including underlying conditions of interest (e.g., Sepsis vs. SIRS or Controls), sample collection time point (e.g., Hospital/ER admission, ICU admission, 24-hours post ICU admission, etc.), tissue type (e.g., whole blood, PBMCs), and various demographic data. A list of all fields of information that collected from each study is summarized in **Supplemental Table 1**, along with detailed descriptions regarding any data standardization protocols that were followed.

### Bioinformatic analysis of curated gene sets

To demonstrate the value that users can gain from analyzing gene sets in SeptiSearch, we performed analyses that provided cross-cutting mechanistic insights based on the collected studies in the first version of the database. For example, we evaluated the most highly represented genes, specifically those genes included in the greatest number of gene sets. Furthermore, to indicate their role in sepsis pathogenesis in a pathway-centric view, the most highly represented genes were also analyzed for over-represented/enriched Reactome pathways using the Sigora R package (v3.0.5; 9, 10) since this gene pair pathway methodology limits the appearance of the same genes in multiple overlapping pathways. To illustrate how the database can be used in the context of user-supplied gene expression datasets, an analysis was performed exploring the dysregulation of SeptiSearch gene sets in gene expression data from 82 ICU patients suspected of pulmonary sepsis previously published by our group (4, 11). The over- or under-expression of signatures in individual patients was estimated using the R package Gene Set Variation Analysis (GSVA; v1.42.0), a non-parametric and unsupervised enrichment statistic (12). Specifically, the GSVA method is used to assess differences in the expression of a gene set when compared to all other genes (i.e., genes not in the set) within each sample. GSVA expression statistics and their associations to patient characteristics (i.e., organ dysfunction, blood culture positivity, and eventual mortality) were visualized as a heatmap using the R package ComplexHeatmap (v2.5.3; 13).

## Results

### Exploring the most highly represented genes in the database

**Figure 1:**
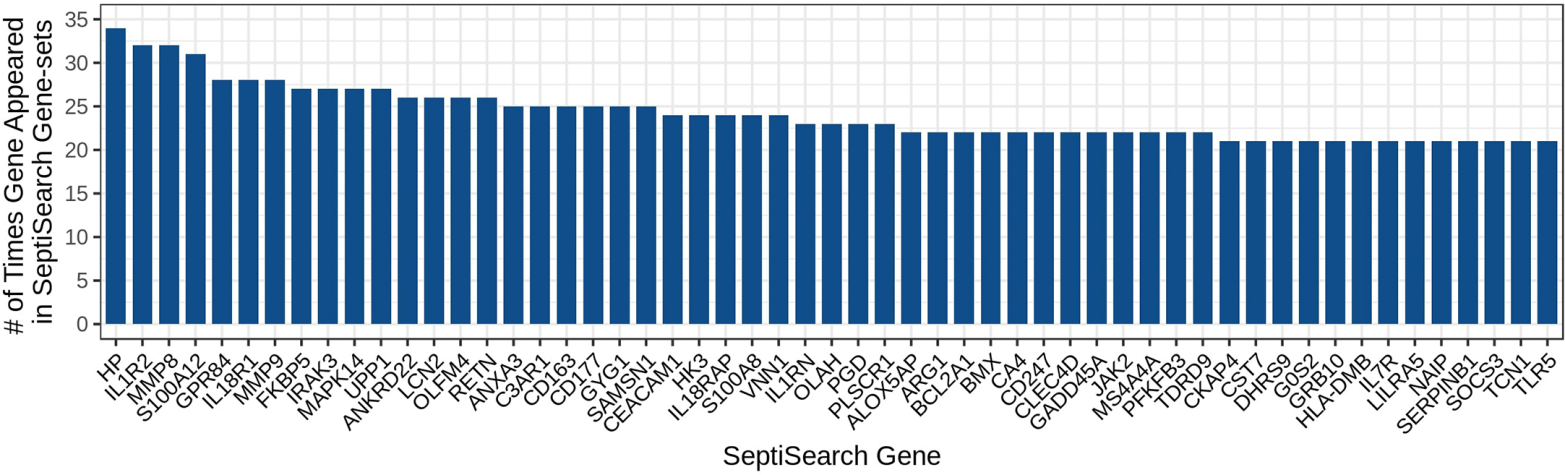
The 50 most highly represented genes amongst the gene sets in the SeptiSearch database. These 50 most represented genes were included in at least 21 gene sets; the occurrence of others can be searched for representation (and which papers identified them) within SeptiSearch, Number.

Given the heterogeneity of sepsis and the diverse conclusions that have been made in individual studies, it would be of great interest to determine whether conclusions made in a given study could be generalized to other studies, and what are the common themes across most similar studies. To enable this type of analysis, gene sets were aggregated into a single database, SeptiSearch. As of November 2022, our group had aggregated results, comprising 20,899 unique genes, from 74 publications that contained 103 sepsis-associated gene sets (**Supplemental Table 2**). We initially assessed which genes were included in multiple publications, indicating genes (or their protein products) strongly implicated in sepsis pathogenesis. The 50 most common genes are identified in **Figure 1**.

Notably, the most highly identified gene associated with sepsis, Haptoglobin (*HP*) an acute-phase marker of red blood cell (RBC) destruction, was found in only 34 of the 103 gene sets (33.0%) surveyed, perhaps reflecting the variability in time of sampling during a patient’s disease, patient age (neonate/pediatric/adult/senior) and other experimental variables, as well as the rather low accuracy methodology (microarrays) used for almost 60% of studies. Intriguingly increased plasma levels of haptoglobin measured early in sepsis are associated with decreased in-hospital mortality so the differential appearance of this gene may also be specific to particular subsets of patients and timing, stressing the importance of metadata associations. A full table of genes represented in at least 10 gene sets is provided in **Supplemental Table 3**.

In addition, most publications provided lists of DE genes rather than utilizing statistical methods to demonstrate significant associations with particular features of sepsis. Thus, the overall DE gene sets obtained in each publication were unique to that publication, with overlaps between publications for specific genes. The 50 most frequently described genes (identified in ≥21/103 gene sets) included Haptoglobin (*HP*), Interleukin 1 Receptor Type 2 (*IL1R2*), Matrix Metallopeptidase 8 and 9 (*MMP8/9*), S100 Calcium Binding Protein A12 (*S100A12*), G Protein-Coupled Receptor 84 (*GPR84*), Interleukin 18 receptor 1 (*IL18R1*), FKBP Prolyl Isomerase 5 (*FKBP5*), Interleukin 1 Receptor Associated Kinase 3 (*IRAK3*), Mitogen-activated Protein Kinase 14 (*MAPK14*), and Uridine Phosphorylase 1 (*UPP1*). Several of these genes are well-described cytokines and chemokines and their receptors.

**Figure 2:**
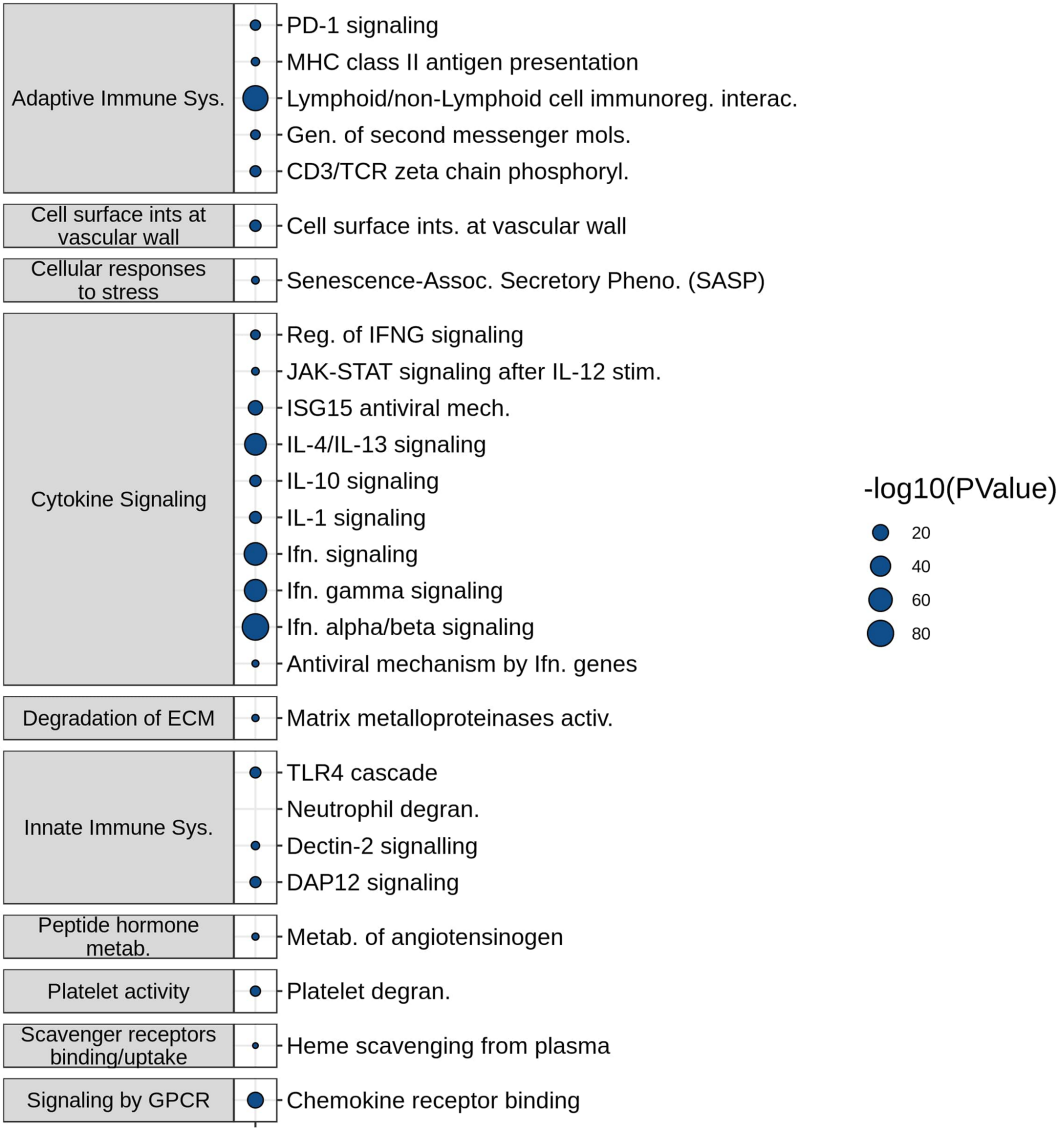
Functional characterization of frequently appearing SeptiSearch genes. The most frequently identified genes in the database were used for Reactome pathway over-representation analysis. For brevity the over-represented pathways shown here displayed p values ≤ 0.001. All over-represented pathways with p values ≤ 0.05 are shown in **Supplemental Table 4**.

The molecular mechanisms represented by the most frequently described DE genes in the SeptiSearch database were deciphered using Reactome pathway over-representation analysis (**Figure 2**). Significantly overrepresented pathways included several immune-related processes, namely Innate and Adaptive immune system, Cytokine signaling, and Platelet-related. These included Neutrophil degranulation, PD-1 signaling, Interferon-α/β signaling, downstream T-cell receptor (TCR) signaling, generation of second messenger molecules, IL-4 and -13 signaling, and IL-10 signaling among others. A complete table over-represented pathway (P value ≤ 0.05) is presented in **Supplemental Table 4**.

Neutrophil degranulation, the most significantly (by p-value) enriched pathway, was of particular interest, given that it has been implicated in the pathogenesis of sepsis organ dysfunction and in particular subsets of patients/endotypes (4, 14). The neutrophil to lymphocyte ratio is well considered as a predictive biomarker of sepsis severity (14), although the enrichment of the neutrophil degranulation pathway does not necessarily imply neutrophil proportions are also elevated. Anti-inflammatory IL-10 cytokine signaling pathway was over-represented, which is a well characterized mediator of immunosuppression in severe sepsis (15), and IL-4/IL-13 pathways associated with M2 (and reprogrammed) macrophages that have been associated with sepsis (16).

Although not the current focus of this database, we also found seven publications using metabolomics that characterized sepsis patients. **Supplemental Figure 1** demonstrates the most highly represented metabolites, namely acetate, citrate, and glucose (included in >4 metabolite-sets). A future direction would be to more extensively curate studies involving metabolomics, proteomics and/or epigenetics to characterize sepsis patients.

### Exploring the gene sets in a previously published cohort of early sepsis patients

To further highlight how the gene sets can be informative to prospective users of SeptiSearch, we examined the expression of the individual gene sets in patient gene expression data from a cohort previously published by our group (4). This included 82 patients suspected of pulmonary sepsis recruited within the first day of ICU admission, for whom whole blood was characterized by RNA-Seq. Participants in this study were adults with written informed consent provided by themselves or by first-degree relatives in cases where the patient was unable to consent. We specifically sought to identify gene sets that were most associated with patient characteristics of interest within the two cohorts, namely organ dysfunction scores and eventual mortality. It is important to note that the universal applicability of proposed diagnostic and prognostic tools (e.g., clinical scores and biomarkers) has not been widely examined. Thus, examining these cohorts in the context of SeptiSearch gene sets may suggest novel opportunities for biomarkers/signatures and understanding early sepsis pathogenesis.

**Figure 3:**
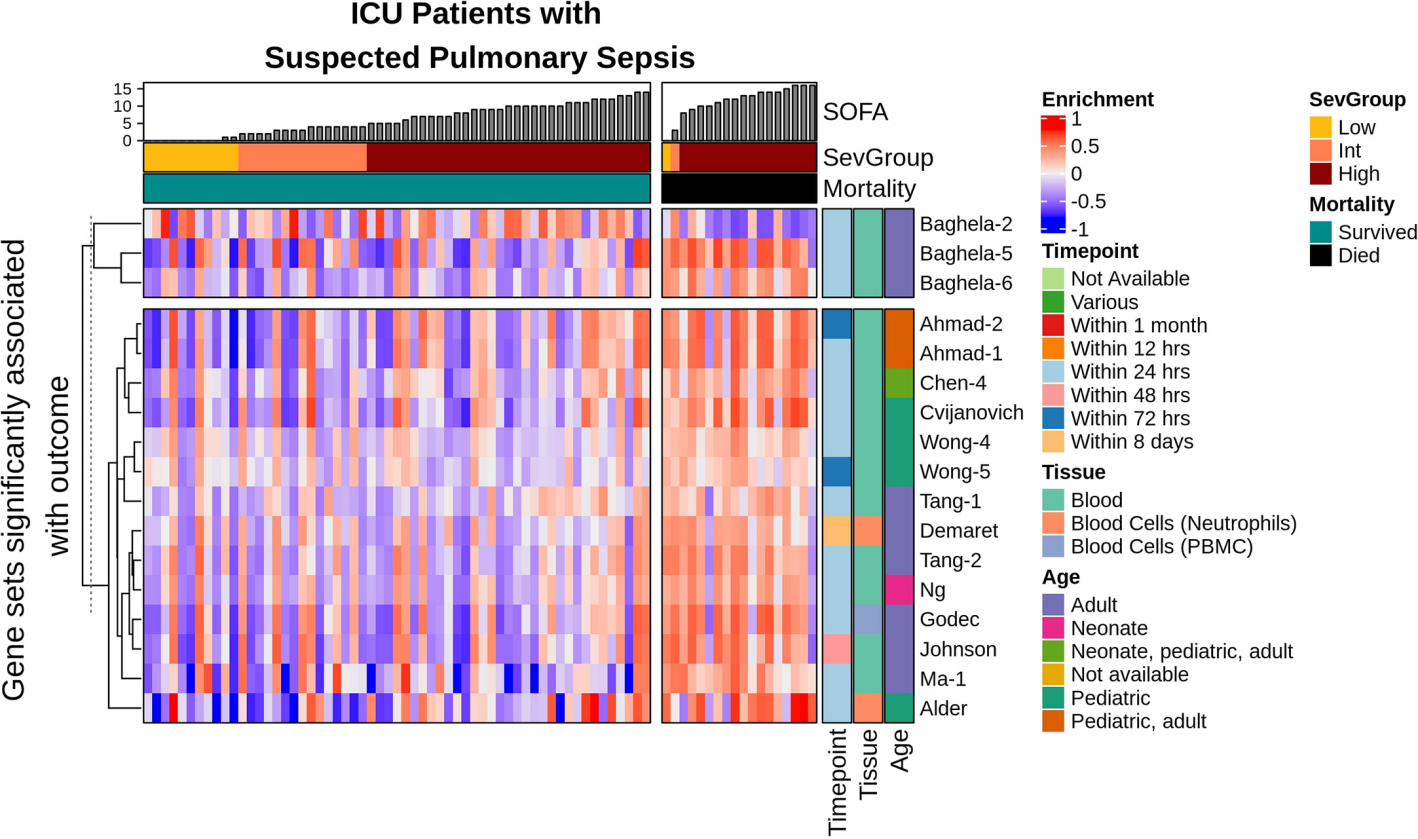
Enrichment of expression of SeptiSearch gene sets significantly associated with eventual mortality in ICU patients with suspected pulmonary sepsis. There were 17 gene sets significantly associated with eventual mortality in ICU patients (shown on y-axis). Most gene sets were strongly up-regulated (red bars) or down-regulated (blue/purple bars). The significant gene sets previously published by Baghela et al. (4) were separated and compared to other gene sets to show that expression trends were largely similar amongst the indicated groups, suggesting coordinate sepsis-dependent expression of the genes making up these individual gene sets. Gene set metadata is explained in **Supplemental Table 1**.

Patient-level enrichment scores for each gene set was estimated using GSVA. Associations between gene set enrichment scores and Sequential Organ Failure Assessment (SOFA, a surrogate for severity) and/or mortality in the two cohorts using linear regression (**Supplemental Figure 2**). Severity groups were characterized as encompassing patients progressing to either High (24 h SOFA scores ≥5), Intermediate (SOFA ≥2; <5), or Low (SOFA <2) severity. Eight gene sets (7.8% of all gene sets) were significantly associated (p value ≤0.01 using linear regression) with patients grouped according to SOFA scores/organ dysfunction in ICU, including one previously published by our group (Baghela-7). Interestingly, there were several more gene sets associated with eventual mortality, as opposed to SOFA severity groups (17, representing 16.5% of all gene sets). **Figure 3** shows how each of the mortality-associated gene sets mapped to the gene expression of individual patients, and is depicted as a heatmap with patient characteristics/outcomes and gene set metadata included to emphasize patterns. Indeed the 17 gene sets that were significantly associated with eventual mortality were generally up-regulated in patients who died. It is visually evident that the expression of many gene sets increased (enrichment indicated by increasing red colour) in patients who eventually succumbed as seen from left to right. There was also one down-regulated gene set among patients who died (i.e., Baghela-2) which captured the opposite relationship.

Comparing the expression of our gene sets (i.e., Baghela-2, 5, 6), derived based on data from these cohorts, with other gene sets based on completely distinct cohorts, it was observed that the general expression trends were similar, with the same individual patients showing increased/decreased expression of all gene sets (strong bands of blue or red running from top to bottom). This indicated that many of the signatures were consistently and coordinately expressed in individual patients, despite the fact that these gene sets were largely composed of different collections of genes. Accordingly, these gene sets are likely mechanistically connected to sepsis and in some cases might be co-regulated or co-expressed via related processes, and might collectively be useful to elucidate functions important in sepsis. It is important to note that the majority of the studies curated originated from blood studies and adult cohorts, which likely involve genes different from those relevant to other organs and pediatric cohorts. Although not demonstrated here, metadata pertaining to gene sets (i.e., age group, tissue type, sampling timepoint) is stored in the SeptiSearch database and can also be used to focus more precisely on user-relevant gene sets. Furthermore, additional patient metadata in user studies, such as the use of immunomodulatory therapies, demographics, genetic background, comorbidities, infection type (i.e., bacterial, viral, fungal), and other measures of patient severity (e.g., APACHE II, SIRS scores) can be used to identify gene sets most relevant to patient groups.

The SeptiSearch database can also be used to assess newly-discovered signatures for their novelty/uniqueness and overlap with previously-discovered gene sets/signatures in sepsis transcriptomic studies. An illustration of this is provided in **Table 1**, where we examined the overlap of our endotype and severe sepsis signatures (4) with those in SeptiSearch. This demonstrated that, although these previously-described signatures are unique, several included frequently identified genes (e.g., **Supplemental Figure 3**; **Supplemental Table 5**). Between 27 and 53% of the genes comprising the severity and mortality signatures were identified in 10 or more separate gene sets within the SeptiSearch database (**Table 1**; **Supplemental Table 5**), indicating that these signature genes were identified in a broad range of patient cohorts. This behaviour was not expected for the 5 identified endotypes that comprise 2 severe endotypes, NPS and INF, and 3 milder endotypes, IFN, IHD and ADA, with IFN having been potentially associated with viral-mediated sepsis. Intriguingly, however, 63.5% of genes associated with the most severe NPS (neutrophilic-suppressive) endotype, were found 10 or more times in SeptiSearch. This is likely because sepsis studies tend to investigate more severely afflicted patients (with NPS exhibiting 45% mortality in the ICU; 4), and may explain why neutrophil degranulation was the leading pathway identified in SeptiSearch gene sets (**Figure 2**). The next most commonly identified set of endotype-specific genes (28.6%) belonged to the IFN endotype, likely reflecting viral infections as a common underlying etiology. In contrast the INF, IHD and ADA endotype markers were far less common (9.0-12.0%). This behaviour is what we at first expected since by definition each endotype reflects only a subset of patients (averaging 20% of patients), and their characterization has been a relatively recent clinical observation. Intriguingly, sepsis was once considered a hyperinflammatory syndrome (involving an initial cytokine storm) and the INF/inflammatory endotype was indeed associated with high severity; but interestingly, pathways associated with inflammation were not commonly represented in SeptiSearch gene sets (**Figure 1**).

**Table 1:** The overlap in signature genes identified in Baghela et al. (4) with those in SeptiSearch.

Signature Name	SeptiSearch Gene set Label	Signature Size	% in well-represented SeptiSearch genes
Severe Sepsis Signatures			
Cellular Reprogramming (CR)	Pena	99	27.3%
Mortality	Baghela-6	38	52.6%
Organ Dysfunction	Baghela-7	52	51.9%
Endotype Signatures			
Adaptive (ADA)	Baghela-1	200	12.0%
Interferon (IFN)	Baghela-2	182	28.6%
Innate Host Defence (IHD)	Baghela-3	200	9.5%
Inflammatory (INF)	Baghela-4	200	9%
Neutrophilic-Suppressive (NPS)	Baghela-5	200	63.5%

Here, we were interested in the overlap of our signatures to ones which were well-represented in the SeptiSearch database (i.e., those genes appearing in ≥10 gene sets). For example, 12.0% of genes comprising the Adaptive endotype signature are well-represented in the SeptiSearch database. Full details are provided in **Supplemental Table 5.**

Beyond assessing the association of gene set enrichment with variables of interest, any of the gene sets in the database or the user’s own data can be subjected to over-representation analysis together with pathway analysis (from the databases Reactome and EnrichR/MSigDB) to determine functions/mechanisms associated with DE gene lists.

### Development of a web application

A web application was created using the Shiny framework in R, to make the gene sets collected in the database freely and conveniently available. The SeptiSearch web app, available at https://septisearch.ca, is an open-source, multi-functional app that presents the gene sets described herein, including the experimental conditions used for their original discovery and associated key metadata (e.g. tissue, time points, patient groups, etc.). The “Explore the Database” tab provides a list of all curated gene sets, the genes within the gene set, relevant metadata, and links to the original publication. Users can analyze summary statistics of all genes/proteins in SeptiSearch through the “Visualize the Database” tab. Here users can filter based on various criteria such as cohort age group, timepoint, tissue, etc. In addition to providing easy-to-use access to the curated sepsis gene sets, additional functionality was added, including over-representation analyses of the curated gene sets or a user-selected gene list (available through the “Perform Pathway Enrichment” tab), and gene set variation analysis (using GSVA) of the gene sets in user-uploaded gene expression data (available through the “Test for Enriched Sepsis Gene Sets” tab). SeptiSearch was designed to be used by biologists and researchers without prior bioinformatics experience, providing members of the sepsis community the tools needed to leverage and explore the database. An online tutorial (https://hancockinformatics.github.io/SeptiSearch/) detailing the use of SeptiSearch is made available, and further details can be found on the SeptiSearch “About” page or the app’s GitHub repository (https://github.com/hancockinformatics/SeptiSearch), where all of the code is available under the GPL-3.0 license.

## Discussion

Here we described the development of SeptiSearch, a manually curated database of sepsis gene sets and signatures aggregating molecular insights from 74 previously published transcriptomics studies. The SeptiSearch database includes 103 individual gene sets comprising those genes associated with sepsis pathogenesis and/or outcomes. In this publication, we have described and explored the database to determine biological mechanisms enriched amongst frequently described genes and highlight how the gene sets can be used to explore user-specific gene expression studies.

The database can serve many purposes in the transcriptomic analysis of sepsis patients and may be extended to related immunological syndromes. For example, we showed biological mechanisms/pathways over-represented in the most frequently represented genes, strengthening their association with sepsis pathogenesis. A notable Reactome pathway that arose in these analyses was Neutrophil degranulation, which has been highlighted in some publications related to the study of sepsis pathogenesis and severity (4, 17). Consistent with the identification of this pathway is the unusual (amongst endotypes) representation (63.5%), amongst the most commonly identified genes in sepsis transcriptomic studies, of the 200 genes that were uniquely differentially expressed in the most severe Neutrophilic Suppressive (NPS) endotype. Several studies have shown that neutrophil dysfunction is associated with an increased risk of secondary infections, a prominent feature of sepsis-induced immunosuppression (18). Interestingly, this pathway and related neutrophil activation signatures have also been shown to be dysregulated in severe Covid-19 infections and accordingly proposed as prognostic biomarkers (19, 20). While neutrophil degranulation is one notable pathway we observed, several other pathways were enriched (e.g., IL-10, PD-1 signaling, etc.), and can be further scrutinized for their role in sepsis and specifically targeted, e.g., IL-10 signaling inhibitors (21) and checkpoint inhibitors of PD-1 signaling (22). Conversely specific hubs in the NPS endotype can be inhibited with repurposed drugs (11).

Gene expression biomarkers offer a promising solution to predict progression to severe sepsis in patients (and inform appropriate therapeutic interventions). Accordingly, SeptiSearch gene sets can provide a starting point for discovering novel predictive biomarkers/signatures. We show that the SeptiSearch gene sets can be measured and assessed for dysregulation in user-specific gene expression profiles. When analyzing early sepsis patients’ gene expression profiles, we showed that particular gene sets were significantly associated with patient SOFA scores and eventual mortality. Significant gene sets can be further analyzed for function and be explored for use as predictive signatures. The database can also provide researchers with more relevant gene sets for enrichment and overrepresentation analysis in their gene expression studies. Importantly, these sets may better capture sepsis-specific immunological processes compared to commonly used function-based databases like Reactome, KEGG, and GO. Liu et al. (23) addressed this hypothesis when they showed that data-derived signatures from leukocytes better detected the presence of benchmarked immunological processes in gene expression data when compared to GO terms.

The curation of gene sets in SeptiSearch also highlighted an interesting aspect of patient transcriptomics studies. The gene sets identified are substantially different between studies and cohorts, despite being established with the same types of patients. This is likely due to differences in variables such as assay technologies used (especially relevant to less accurate microarray technologies), comparators (i.e., the use as non-sepsis comparators of surgical controls, healthy individuals, SIRS patients, etc.), time of collection of data relative to hospitalization (usually ICU patients), definitions of sepsis (e.g., SIRS plus infection; Sepsis-2, Sepsis-3, bloodstream infection), and normalization, as well as the specific mixture of endotypes making up a study’s cohort (with apparent skewing to the NPS endotype). Also, when predictive signatures were proposed (which was infrequent), technical aspects such as statistical/machine learning methods for feature selection, and the prediction algorithms used varied. Interestingly, although the basic details differ (e.g., the specific genes within the signatures), their most prominent represented pathways overlapped when analyzed in aggregate (e.g., Neutrophil degranulation). Thus, although different molecules have been identified or selected in these various studies, some may still be involved in the same cellular processes.

SeptiSearch is a biologist-friendly resource that provides members of the sepsis research community with the bioinformatics tools needed to explore and make use of the curated gene sets in gene expression datasets. Further directions to make SeptiSearch more valuable would be more extensively curating sepsis-related publications, which includes exploring other databases of biomedical and life sciences literature (e.x., Cochrane Library, Web of Science, or EMBASE) for relevant publications. Another notable future direction is to more extensively curate studies involving metabolomics, proteomics, epigenetics, and other omic modalities that are used to characterize sepsis patients.

## Data availability statement

The original contributions presented in the study are included in the article/**Supplementary Material**. Further inquiries can be directed to the corresponding author.

## Ethics statement

The studies involving human participants were reviewed and approved by The University of British Columbia. The patients/participants provided their written informed consent to participate in this study.

## Author contributions

AB and RH conceived the study. AB, JT, and TB curated gene sets from the literature. TB created the SeptiSearch Shiny web application. AB performed bioinformatics analysis. AB, BD, and RH contributed to the interpretation of data. AB and RH drafted the manuscript. All authors contributed to the article and approved the submitted version.
