# Supplementary material for: Facilitating systems-level analyses of all-cause and Covid-mediated sepsis through SeptiSearch, a manually-curated compendium of dysregulated gene sets
Source: Front Immunol. 2023 May 26;14:1135859. doi: 10.3389/fimmu.2023.1135859 (PMC10250596; doi:10.3389/fimmu.2023.1135859)
Supplement: Supplementary file 1 [file DataSheet_1.docx]

**Facilitating systems-level analyses of all-cause and Covid-mediated sepsis through SeptiSearch, a manually-curated compendium of dysregulated gene sets.**

Arjun S. Baghela, Jasmine Tam, Travis M. Blimkie, Bhavjinder K. Dhillon, Robert E.W. Hancock*

Centre for Microbial Diseases and Immunity Research, University of British Colombia, 232-2259 Lower Mall, Vancouver V6T 1Z4, Canada’

* Corresponding author: email [bob@hancocklab.com](mailto:bob@hancocklab.com); Tel 604 822 2682.

**SUPPLEMENTAL MATERIAL**


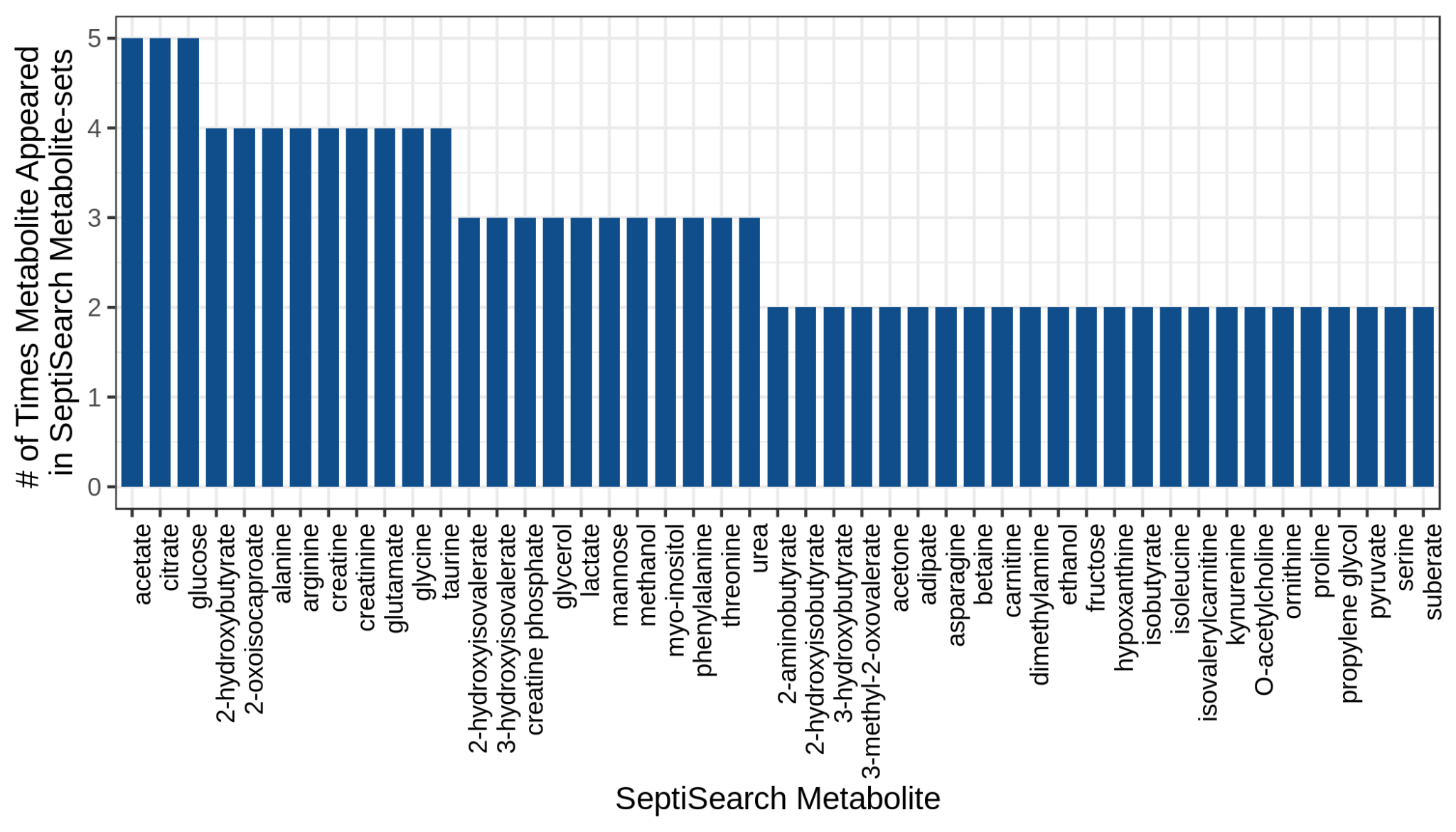


**Supplemental Figure 1 – Most highly identified metabolites amongst gene sets in the database.** A future direction of the SeptiSearch is including metabolomics and proteomics studies curation efforts.

**
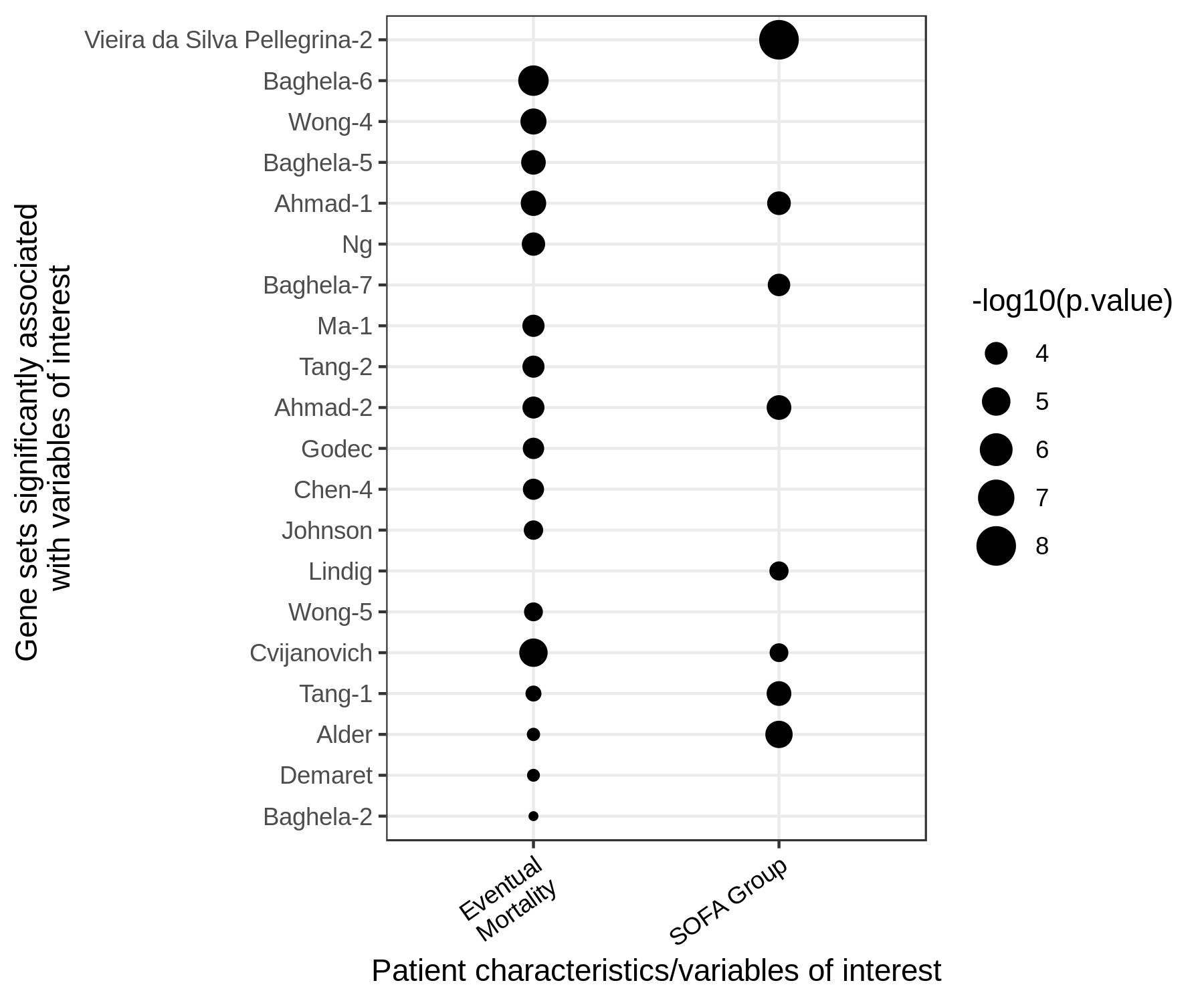
**

**Supplemental Figure 2 – Significant associations between gene sets and 24-hour SOFA score or eventual mortality in a gene expression dataset.** This study included 82 ICU patients where blood was taken within 24-hours of ICU admission for RNA-Seq (Baghela et al, 2022). Gene set expression was captured for each listed data-set by GSVA enrichment scores. Association was assessed using linear regression, with the outcomes of interest used as the independent variable and the GSVA enrichment scores as the response variable. Significant associations displayed P values ≤ 0.01. There were 8 gene sets associated with SOFA scores and 17 gene sets associated with eventual mortality in ICU patients. Five of these gene sets overlapped with each other (i.e., Ahmad-1, Ahmad-2, Cvijanovich, Tang-1, and Alder).


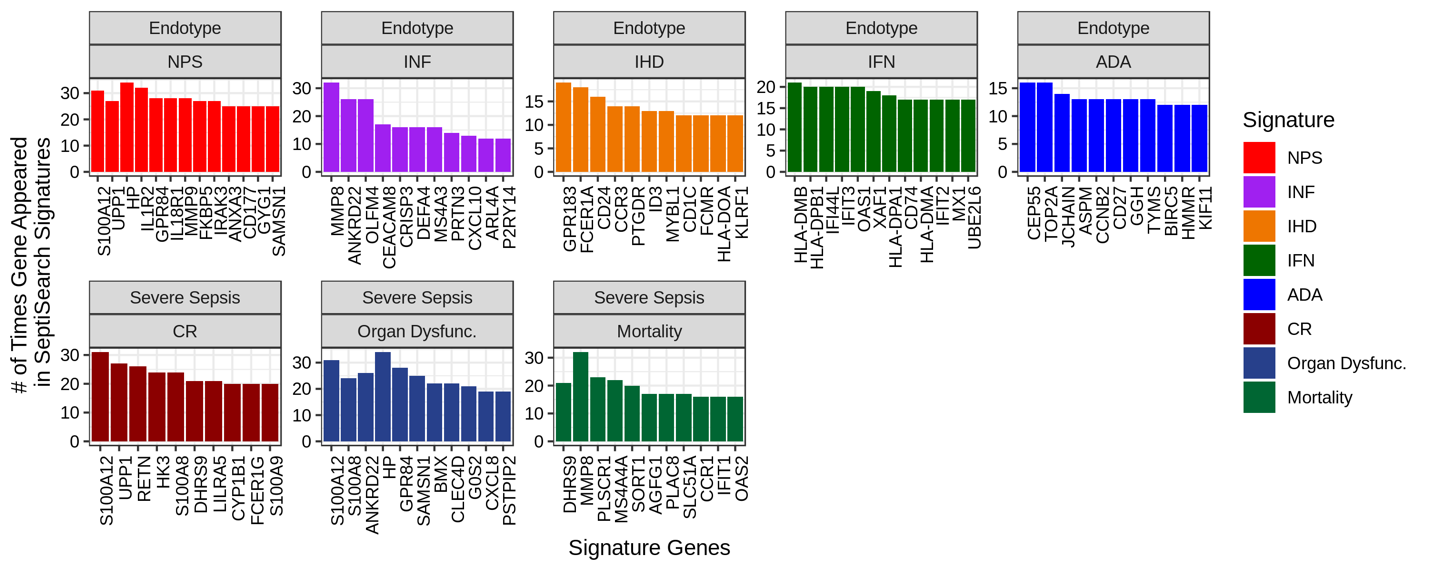


**Supplemental Figure 3 – The gene sets (signatures) described in Baghela et al. (2022) were comprised of very well-represented SeptiSearch genes.** Shown here are the top ten most highly represented genes in each gene set.

**Supplemental Table 1 - Fields of information that was collected from each study.** Detailed descriptions regarding standardization protocols are provided. Other study meta-data was also collected when available, including case condition (e.g., sepsis, severe sepsis, endotype), control condition (e.g., healthy control), cohort severity, cohort demographics (i.e., Male/Female ratio), transcriptomics platform (e.g., RNA-Seq or microarray platform), statistical methods used, sampling timepoint. Missing data was codified as “NA” in instances where data was not provided in the text or not readily available. This data is provided in the downloadable file available at https://github.com/hancockinformatics/SeptiSearch.

| **Data Curated** | **Description** |
| --- | --- |
| Author (study label) | The study label is indicated by the author's name; multiple gene sets published by the same author are coded with an additional numeric label (i.e., -1, -2, etc.). |
| PMID | Study PMID. |
| Tissue | Septisearch studies included gene sets originating from several tissue/primary cell types. These were labelled as following: (1) Blood (or whole blood); (2) Blood Cells (which included peripheral blood mononuclear cell [PBMC], primary cells isolated from blood, such as neutrophils, or leukocytes); (3) Lung Cells (which included lung tissues, Bronchioalveolar Lavage or primary cells isolated from the respiratory systems, including normal Human Bronchial Epithelial [NHBE] cells; (4) Other (which included Nasopharyngeal cells from swabs). |
| Age Group | Age group indicates whether the gene set originated from adult (including senior), pediatric, or neonatal patient populations. |
| Covid status | Covid status indicates whether the study analyzed patients with Covid-19 (COVID) or other types of sepsis/all-cause sepsis (Non-COVID). |
| Type of Signature | The type of signature indicates whether the gene set represents a list of differentially expressed genes or a gene set specifically referred to as a signature by the authors (this typically involved the selection of differentially expressed genes for predictive models of disease or clinical outcomes). |
| Number of genes | The number of genes included in each gene set. |

**Supplemental Table 2 – Curated SeptiSearch gene sets and their respective publication.** In total, 103 signatures were aggregated. DA = Differentially Abundant (Expressed).

| **Author (study label)** | **PMID** | **Tissue** | **Age group** | **Covid status** | **Type of signature** | **Number of genes** |
| --- | --- | --- | --- | --- | --- | --- |
| Abbas | 32859206 | Blood | Pediatric | Non-COVID | Signature | 11 |
| Ahmad-1 | 31817302 | Blood | Pediatric, adult | Non-COVID | Signature | 81 |
| Ahmad-2 | 31817302 | Blood | Pediatric, adult | Non-COVID | Signature | 65 |
| Alder | 27635771 | Blood Cells (Neutrophils) | Pediatric | Non-COVID | Signature | 5 |
| Almansa-1 | 25557485 | Blood | Adult | Non-COVID | DA | 1975 |
| Almansa-2 | 25557485 | Blood | Adult | Non-COVID | Signature | 72 |
| Arunachalam | 32788292 | Blood Cells (PBMC) | Adult | COVID | DA | 2290 |
| Aschenbrenner-1 | 33441124 | Blood | Adult | COVID | DA | 3205 |
| Aschenbrenner-2 | 33441124 | Blood | Adult | COVID | DA | 1479 |
| Aschenbrenner-3 | 33441124 | Blood | Adult | COVID | DA | 5033 |
| Aschenbrenner-4 | 33441124 | Blood | Adult | COVID | DA | 755 |
| Baghela-1 | 35027333 | Blood | Adult | Non-COVID | DA | 200 |
| Baghela-2 | 35027333 | Blood | Adult | Non-COVID | DA | 182 |
| Baghela-3 | 35027333 | Blood | Adult | Non-COVID | DA | 200 |
| Baghela-4 | 35027333 | Blood | Adult | Non-COVID | DA | 200 |
| Baghela-5 | 35027333 | Blood | Adult | Non-COVID | DA | 200 |
| Baghela-6 | 35027333 | Blood | Adult | Non-COVID | Signature | 38 |
| Baghela-7 | 35027333 | Blood | Adult | Non-COVID | Signature | 52 |
| Banerjee | 33692779 | Blood | Pediatric | Non-COVID | Signature | 8 |
| Bauer | 27211554 | Blood | Adult | Non-COVID | Signature | 7 |
| Blanco-Melo-1 | 32416070 | Lung Cells (NHBE) | Adult | COVID | DA | 86 |
| Blanco-Melo-2 | 32416070 | Blood | Adult | COVID | DA | 3447 |
| Burnham-1 | 28036233 | Blood Cells (Leukocytes) | Adult | Non-COVID | DA | 4193 |
| Burnham-2 | 28036233 | Blood Cells (Leukocytes) | Adult | Non-COVID | DA | 267 |
| Burnham-3 | 28036233 | Blood Cells (Leukocytes) | Adult | Non-COVID | Signature | 920 |
| Cazalis | 26215705 | Blood | Adult | Non-COVID | Signature | 46 |
| Cernada | 24709930 | Blood | Neonate | Non-COVID | DA | 439 |
| Chen-1 | 24668300 | Blood | Neonate | Non-COVID | Signature | 82 |
| Chen-2 | 32217835 | Blood | Adult | COVID | Signature | 6 |
| Chen-3 | 32217835 | Blood | Adult | COVID | Signature | 3 |
| Chen-4 | 31093495 | Blood | Neonate, pediatric, adult | Non-COVID | Signature | 64 |
| Cvijanovich | 18460642 | Blood | Pediatric | Non-COVID | Signature | 40 |
| Davenport | 26917434 | Blood | Adult | Non-COVID | Signature | 3055 |
| Demaret | 27568821 | Blood Cells (Neutrophils) | Adult | Non-COVID | DA | 460 |
| Desai | 33298930 | Lung Cells (Tissue) | Adult | COVID | DA | 6057 |
| George | 33883570 | Lung Cells (BALF) | Adult | COVID | DA | 31 |
| Godec | 26795250 | Blood Cells (PBMC) | Adult | Non-COVID | Signature | 667 |
| Hadjadj | 32661059 | Blood | Adult | COVID | DA | 6 |
| Han | 30297806 | Blood | Adult | Non-COVID | Signature | 10 |
| Howrylak | 19174476 | Blood | Adult | Non-COVID | Signature | 8 |
| Huang-1 | 31986264 | Blood | Adult | COVID | DA | 17 |
| Huang-2 | 31986264 | Blood | Adult | COVID | DA | 8 |
| Johnson | 17414611 | Blood | Adult | Non-COVID | Signature | 55 |
| Kangelaris | 25795726 | Blood | Adult | Non-COVID | Signature | 6 |
| Karki | 33278357 | Blood | Adult | COVID | Signature | 7 |
| Khan | 31280495 | Blood | Adult | Non-COVID | Signature | 40 |
| Labiad | 29885947 | Blood Cells (PBMC) | Adult | Non-COVID | Signature | 11 |
| Lee-1 | 32651212 | Blood Cells (PBMC) | Adult | Non-COVID | DA | 794 |
| Lee-2 | 32651212 | Blood Cells (PBMC) | Adult | COVID | DA | 169 |
| Lee-3 | 32651212 | Blood Cells (PBMC) | Adult | COVID | DA | 1076 |
| Leite | 31396396 | Blood | Adult | Non-COVID | Signature | 4 |
| Lieberman-1 | 32898168 | Other Cells (Nasopharyngeal) | Adult | COVID | Signature | 83 |
| Lieberman-2 | 32898168 | Other Cells (Nasopharyngeal) | Adult | COVID | Signature | 363 |
| Lindig | 23684387 | Blood | Pediatric, adult | Non-COVID | Signature | 14 |
| Ma-1 | 24146790 | Blood | Adult | Non-COVID | Signature | 5 |
| Ma-2 | 24146790 | Blood | Adult | Non-COVID | Signature | 2 |
| Ma-3 | 26063982 | Blood | Pediatric, adult | Non-COVID | Signature | 30 |
| Madsen-Bouterse | 20059468 | Blood | Pediatric, adult | Non-COVID | DA | 25 |
| Maslove | 23036193 | Blood Cells (Neutrophils) | Adult | Non-COVID | Signature | 167 |
| McHugh | 26645559 | Blood | Adult | Non-COVID | Signature | 4 |
| Mohammed | 31375728 | Blood | Pediatric | Non-COVID | Signature | 16 |
| Mommert | 32188504 | Blood | Adult | Non-COVID | Signature | 193 |
| Ng | 32479514 | Blood | Neonate | Non-COVID | DA | 1263 |
| Pankla-1 | 19903332 | Blood | Adult | Non-COVID | Signature | 16 |
| Pankla-2 | 19903332 | Blood | Adult | Non-COVID | Signature | 37 |
| Pena | 25685830 | Blood | Adult | Non-COVID | Signature | 99 |
| Prucha | 15201698 | Blood | Adult | Non-COVID | Signature | 50 |
| Qin | 32161940 | Blood | Adult | COVID | DA | 11 |
| Reyes | 32066974 | Blood Cells (PBMC) | Adult | Non-COVID | Signature | 50 |
| Sahoo-1 | 32995790 | Blood | NA | COVID | Signature | 166 |
| Sahoo-2 | 32995790 | Blood | NA | COVID | Signature | 20 |
| Schaack | 29920518 | Blood | Adult | Non-COVID | Signature | 5 |
| Scicluna | 28864056 | Blood | Pediatric, adult | Non-COVID | Signature | 140 |
| Shen | 24668717 | Blood | Adult | Non-COVID | Signature | 14 |
| Sousa | 26276539 | Blood | Adult | Non-COVID | DA | 3781 |
| Sweeney-1 | 25972003 | Blood | Pediatric | Non-COVID | Signature | 11 |
| Sweeney-2 | 29449546 | Blood | Neonate, pediatric, adult | Non-COVID | Signature | 58 |
| Sweeney-3 | 29537985 | Blood | Pediatric, adult | Non-COVID | Signature | 33 |
| Tabone | 30671061 | Blood | Adult | Non-COVID | Signature | 56 |
| Talwar-1 | 16403844 | Blood Cells (PBMC) | Adult | Non-COVID | DA | 814 |
| Talwar-2 | 16403844 | Blood | Adult | Non-COVID | Signature | 173 |
| Tang-1 | 18379237 | Blood | Adult | Non-COVID | Signature | 93 |
| Tang-2 | 19237892 | Blood | Adult | Non-COVID | Signature | 123 |
| Tang-3 | 17575094 | Blood | Adult | Non-COVID | Signature | 50 |
| Thair-1 | 33437935 | Blood | Neonate, pediatric, adult | COVID | Signature | 413 |
| Thair-2 | 33437935 | Blood | Adult | COVID | Signature | 1998 |
| Toraih | 33289122 | Lung Cells (NHBE) | Adult | COVID | Signature | 161 |
| Vieira da Silva Pellegrina-1 | 26047321 | Blood | Adult | Non-COVID | Signature | 7 |
| Vieira da Silva Pellegrina-2 | 26047321 | Blood | Adult | Non-COVID | Signature | 16 |
| Wang | 27090785 | Blood | Adult | Non-COVID | Signature | 20 |
| Wong-1 | 25489881 | Blood | Pediatric | Non-COVID | Signature | 100 |
| Wong-2 | 17374846 | Blood | Pediatric | Non-COVID | Signature | 55 |
| Wong-3 | 19325468 | Blood | Pediatric | Non-COVID | DA | 1378 |
| Wong-4 | 19325468 | Blood | Pediatric | Non-COVID | DA | 606 |
| Wong-5 | 19325468 | Blood | Pediatric | Non-COVID | DA | 1574 |
| Wong-6 | 19624809 | Blood | Pediatric | Non-COVID | Signature | 44 |
| Wong-7 | 28324661 | Blood | Pediatric | Non-COVID | Signature | 18 |
| Wong-8 | 21705885 | Blood | Pediatric | Non-COVID | Signature | 90 |
| Wynn-1 | 26052715 | Blood | Neonate | Non-COVID | Signature | 30 |
| Wynn-2 | 26052715 | Blood | Neonate | Non-COVID | Signature | 32 |
| Xiong-1 | 32228226 | Lung Cells (BALF) | Adult | COVID | DA | 1004 |
| Xiong-2 | 32228226 | Blood Cells (PBMC) | Adult | COVID | DA | 1021 |
| Zhou | 32407669 | Lung Cells (BALF) | Adult | COVID | Signature | 1753 |

**Supplemental Table 3 – Genes represented in at least 10 SeptiSearch gene sets.** This captured a total of 775 genes.

| **Gene set mentions** | **Genes** |
| --- | --- |
| **30-34** | HP, IL1R2, MMP8, S100A12 |
| **25-29** | GPR84, IL18R1, MMP9, FKBP5, IRAK3, MAPK14, UPP1, ANKRD22, LCN2, OLFM4, RETN, ANXA3, C3AR1, CD163, CD177, GYG1, SAMSN1 |
| **20-24** | CEACAM1, HK3, IL18RAP, S100A8, VNN1, IL1RN, OLAH, PGD, PLSCR1, ALOX5AP, ARG1, BCL2A1, BMX, CA4, CD247, CLEC4D, GADD45A, JAK2, MS4A4A, PFKFB3, TDRD9, CKAP4, CST7, DHRS9, G0S2, GRB10, HLA-DMB, IL7R, LILRA5, NAIP, SERPINB1, SOCS3, TCN1, TLR5, ALPL, CR1, CYP1B1, FCER1G, FCGR1A, FGR, GBP1, HLA-DPB1, IFI44L, IFIT3, MGAM, OAS1, S100A9, SORT1 |
| **15-19** | ACSL1, CD3E, CXCL8, ETS2, F5, FCAR, GPR183, ITGAM, LTF, MAP2K6, NLRC4, PFKFB2, PSTPIP2, SLC11A1, SLC2A3, TSPO, XAF1, ZAP70, AUTS2, B4GALT5, C1QB, CD3G, CLEC5A, DACH1, FCER1A, FLOT1, GADD45B, HLA-DPA1, IFITM1, IL10, IL1B, KLRB1, LDHA, LRG1, MCEMP1, PGLYRP1, RGL4, STOM, TLR2, TNFSF10, ZDHHC19, ADM, AGFG1, ASPH, BPI, CD3D, CD74, CEACAM8, CEBPB, DYSF, FAM20A, FPR1, FYN, GALNT14, GBP5, GK, HLA-DMA, IFIT2, IL2RB, IL4R, KCNE1, LCK, LEF1, LIMK2, MAFG, MARCKS, MERTK, MX1, NELL2, NSUN7, OPLAH, OSM, PLAC8, RNASE2, SLC51A, SPTLC2, TCF7, THBS1, TNFAIP6, UBE2L6, ABLIM1, ADAM9, BASP1, BCL11B, CAPG, CCR1, CCR7, CD2, CD24, CD55, CD6, CD63, CDC42EP3, CEP55, CLEC4E, CRISP3, CTSD, DEFA4, ELANE, EPSTI1, FGF13, FOLR3, FPR2, GNLY, IDI1, IER3, IFI27, IFIT1, IFITM3, IL1R1, LGALS1, LMNB1, LRRN3, LTB4R, LY9, MAL, MCTP2, METTL7B, MKNK1, MME, MPO, MS4A3, NFKBIA, OAS2, QSOX1, RAB20, RNASE3, S100P, SERPING1, ST6GALNAC3, STAT1, TBC1D8, TOP2A, WSB1, ACVR1B, ADGRG3, ALOX5, AP3B2, AQP9, ATP9A, AZU1, BATF, BCL11A, BCL6, BST1, CACNA1E, CAMP, CARD6, CD8A, CEACAM6, EMILIN2, EXOSC4, FLOT2, GZMB, GZMM, HGF, HLA-DRA, HPGD, IFI35, IFI6, IL32, IRF7, ISG15, ITK, LY96, METTL9, ORM1, PASK, PCOLCE2, PDE4B, PDGFC, PLXNC1, PPARG, PTEN, RAB27A, SERPINA1, SERPINB2, SLC26A8, SLPI, SPOCK2, STXBP2, TIMP1, UBE2J1, UGCG |
| **10-14** | AIM2, BAZ1A, BCL2, BTN3A2, CCL4, CCL5, CCR3, CD160, CD28, CD82, CD96, CLIC1, CYP19A1, CYSTM1, DAAM2, DDAH2, DRAM1, ECHDC3, EIF4E3, EVL, FAM89A, FBXO6, GCA, GCH1, GZMK, HLA-DRB1, IFIT5, JCHAIN, KCNMA1, KIF1B, KREMEN1, LY6E, MXD1, OAS3, PADI4, PARP9, PGS1, PIK3AP1, PLBD1, PLP2, PRF1, PROK2, PRTN3, PTGDR, PYGL, RASGRP1, RGS2, SAMD3, SELL, SEMA4A, SKAP2, SQOR, STAT4, TGFBI, TLR8, TNFRSF25, TP53I3, TPST1, TRIB1, TRIM22, TXN, ASPM, BCAT1, BTN3A1, C1QC, CARD16, CCNA1, CCNB2, CD14, CD27, CD8B, CDC25B, CFLAR, CLIC3, CNIH4, CSGALNACT2, CX3CR1, CXCL10, DDX58, DIAPH2, ENTPD7, FAM117B, FCGR1B, FES, FFAR2, FFAR3, GAPDH, GAS7, GATA3, GGH, GPR141, GPR160, GZMA, GZMH, HCAR3, HERC5, HIF1A, HIP1, HLA-DQA1, HLA-DQB1, ID3, IFI44, IFIH1, IFNGR1, JUN, KLF12, KLHL3, KLRD1, KLRG1, LAP3, LDLR, LILRA6, LILRB3, LIN7A, LRRN1, MAFF, MARCO, MATK, MGST1, MT2A, MTF1, MYBL1, MYO10, NAMPT, NCR3, NQO2, PAG1, PDCD4, PLAAT4, PLIN3, RPL3, RSAD2, RTP4, RUNX3, S100A11, SAT1, SH3GLB1, SIGLEC5, SIGLEC9, SIPA1L2, SKAP1, SLA, SMPDL3A, THEMIS, TNF, TYMP, TYMS, ZFP36L2, ZNF438, ZNF91, ACER3, ACSL4, ADGRE1, AGTRAP, AHNAK, ANKS1A, ANXA1, ARL4A, ARL4C, ATP11B, BCL3, BIRC3, BIRC5, CD1C, CD5, CD58, CD59, CD79A, CHI3L1, CKS2, CREB5, DENND3, DHRS13, DYRK2, EEF1A1, EIF4G3, ERLIN1, EXT1, FCMR, FGD4, FGL2, FOS, GBP4, GNS, GRN, HLA-DOA, HMMR, HSPA1A, ITGA2B, ITGA7, KIF11, KLHL2, KLRF1, LAT, LBH, LDHB, LPAR6, MDFIC, MEGF9, MTHFD2, MYOF, NCF4, NFIL3, NLRC3, NMI, NOG, NTSR1, OSCAR, P2RY14, PLA2G7, PPP1R3D, RAB13, RAB32, RHOU, RPL15, RPS6KA5, SH2D1A, SLC37A3, SLC39A8, SOS2, SRPK1, SULT1B1, SYTL2, TBC1D4, TLR4, TNFSF13B, TRIM25, UPB1, VASP, VCAN, WDFY3, WIPI1, ZEB2, ABCG1, AFF3, APOL6, ARHGAP24, ATM, ATP6V1C1, ATP8B2, AZI2, B3GNT5, BACH2, BST2, BTBD11, BTN3A3, CACNA2D3, CAMK4, CAPZA1, CASP4, CCNA2, CCNB1, CD274, CD4, CD44, CD69, CDK5RAP2, CDKN3, CEBPD, CLEC10A, CLEC7A, CLIC4, CPA3, CPD, CPEB4, CPVL, CRIP1, CSF3R, CST3, CTSA, CTSG, CXCR6, DOCK10, DOK3, ELL2, ENO1, EOMES, EPAS1, ESYT1, ETS1, EXOC6, FAM102A, FAM110B, FCGR2A, FOSL2, GALNT2, GIMAP7, GLIPR2, GNG5, GOLGA8A, GRINA, HMGB2, HVCN1, IGFBP7, IL10RB, IL1RAP, IL5RA, ITGAX, ITM2A, JAK3, JUNB, KDM6B, KL, KLF6, LAIR1, LDLRAP1, LGALS3BP, LMO2, LPCAT2, LYN, MAFB, MAN1C1, MEF2A, MKI67, MSRA, MYC, MZB1, NCALD, NECAB1, NFE2, NRN1, NT5E, OASL, PADI2, PARP14, PCMT1, PHC2, PHTF1, PI3, PKM, PLEKHA1, PLEKHO1, PNPLA1, POR, PSME2, PTGR1, PTGS2, PTPRC, QPCT, RAB31, RBMS1, RFTN1, ROPN1L, RPL13A, RPL5, SAP30, SGK1, SH3PXD2B, SH3YL1, SIRPA, SLC16A3, SLC1A3, SLC25A6, SLC36A1, SLC40A1, SLC4A7, SLCO4C1, SMARCD3, SPATS2L, SPI1, STMN3, SULF2, SYNE2, TAGAP, TAP1, TC2N, TCTN1, TIFA, TMEM165, TP53I11, TPST2, TRAF5, TRBC1, TTC39C, UBE2H, USB1, VNN2, VSIG4, WARS1, ZBP1, ABCA13, ADGRG1, ALPK1, ANKRD34B, APOL1, ARPC5, BNIP3L, BUB1, CAMK2D, CASP1, CCDC88C, CCR6, CD40LG, CD7, CDKN2D, CFD, CHMP5, CLC, CLEC1B, CMPK2, CTSC, CTSW, CXCL1, CXCR3, CYP27A1, DDIAS, DDX60, DDX60L, DLGAP5, DOCK9, DPY19L3, DSC2, DTX3L, DUSP6, DYNLT1, EEF2, EFHD2, EPHA4, EPHB1, FAR2, FAS, FKBP1A, FNDC3B, GBA, GCLM, GIMAP5, GLRX, GM2A, GNAQ, GPR68, H2AC18, H2BC5, HLA-DRB5, HPR, HSH2D, HSPA1B, ICOS, IFNGR2, IGSF6, IL17RA, IMPA2, INHBA, ITGB2, ITGB7, JMJD6, KIAA0930, KLRC1, KLRK1, LGALS2, LITAF, MAOA, MS4A1, MSRB1, MSRB2, N4BP1, NEXN, NFKBIZ, NIBAN1, NLRP1, NMT2, NUP210, OLR1, OPTN, OTOF, P2RX1, P2RY10, PCGF5, PDE4D, PDSS1, PIK3IP1, PLCG1, PLD1, POLR3E, PRKCH, PRR5L, PTGER4, PTX3, RABGAP1L, RGCC, RHOH, RNF213, RPL10A, RPL12, RPL14, RPS4X, RPS5, RPS6, RPS8, RRAGD, RTN1, S1PR5, SAMD9, SAMD9L, SCPEP1, SET, SH2D1B, SHCBP1, SIRPG, SLC22A4, SLCO4A1, SNRPN, SOD2, SP100, SSH1, STK3, STX11, TACSTD2, TIGIT, TIMP2, TK1, TLR1, TMCO3, TMEM204, TMEM8B, TNFAIP2, TNFAIP3, TOR1B, TRPM2, TSHZ3, UBASH3A, VIM, ZWINT |

**Supplemental Table 4 – All significant pathways identified in the functional characterization of frequently appearing SeptiSearch genes.** The most frequently identified genes (identified in at least 10) were used for Reactome pathway over-representation analysis.

| **Pathway ID** | **P Value** | **Pathway Name** | **Pathway Group** |
| --- | --- | --- | --- |
| R-HSA-6798695 | 0 | Neutrophil degranulation | Innate Immune Sys. |
| R-HSA-909733 | 4.37E-86 | Ifn. alpha/beta signaling | Cytokine Signaling |
| R-HSA-198933 | 1.41E-75 | Lymphoid/non-Lymphoid cell immunoreg. interac. | Adaptive Immune Sys. |
| R-HSA-913531 | 3.79E-58 | Ifn. signaling | Cytokine Signaling |
| R-HSA-877300 | 2.69E-55 | Ifn. gamma signaling | Cytokine Signaling |
| R-HSA-6785807 | 3.78E-52 | IL-4/IL-13 signaling | Cytokine Signaling |
| R-HSA-380108 | 2.36E-23 | Chemokine receptor binding | Signaling by GPCR |
| R-HSA-1169408 | 4.49E-18 | ISG15 antiviral mech. | Cytokine Signaling |
| R-HSA-9020702 | 2.82E-11 | IL-1 signaling | Cytokine Signaling |
| R-HSA-202733 | 6.07E-10 | Cell surface ints. at vascular wall | Cell surface ints at vascular wall |
| R-HSA-6783783 | 6.94E-10 | IL-10 signaling | Cytokine Signaling |
| R-HSA-2424491 | 2.31E-09 | DAP12 signaling | Innate Immune Sys. |
| R-HSA-202427 | 2.74E-09 | CD3/TCR zeta chain phosphoryl. | Adaptive Immune Sys. |
| R-HSA-166016 | 3.31E-09 | TLR4 cascade | Innate Immune Sys. |
| R-HSA-389948 | 6.40E-08 | PD-1 signaling | Adaptive Immune Sys. |
| R-HSA-114608 | 8.29E-08 | Platelet degranulation | Platelet activity |
| R-HSA-202433 | 8.42E-07 | Gen. of second messenger mols. | Adaptive Immune Sys. |
| R-HSA-877312 | 8.88E-07 | Reg. of IFNG signaling | Cytokine Signaling |
| R-HSA-5621480 | 2.75E-05 | Dectin-2 signalling | Innate Immune Sys. |
| R-HSA-2132295 | 2.95E-05 | MHC class II antigen presentation | Adaptive Immune Sys. |
| R-HSA-2559582 | 1.21E-04 | Senescence-Assoc. Secretory Pheno. (SASP) | Cellular responses to stress |
| R-HSA-8950505 | 1.29E-04 | JAK-STAT signaling after IL-12 stim. | Cytokine Signaling |
| R-HSA-1592389 | 2.58E-04 | Matrix metalloproteinases activ. | Degradation of ECM |
| R-HSA-2022377 | 2.85E-04 | Metab. of angiotensinogen | Peptide hormone metab. |
| R-HSA-1169410 | 3.35E-04 | Antiviral mechanism by Ifn. genes | Cytokine Signaling |
| R-HSA-449836 | 3.74E-04 | Other interleukin signaling | Cytokine Signaling |
| R-HSA-2168880 | 7.76E-04 | Heme scavenging from plasma | Scavenger receptors binding/uptake |
| R-HSA-5668599 | 0.001149 | RHO GTPases Activ. NADPH oxidases | Signaling by Rho GTPases |
| R-HSA-2029481 | 0.001227 | FCGR activation | Innate Immune Sys. |
| R-HSA-391160 | 0.002346 | Signal regulatory protein family ints. | Signal regulatory protein family ints. |
| R-HSA-2559583 | 0.002589 | Cellular senescence | Cellular responses to stress |
| R-HSA-446652 | 0.002878 | IL-1 family signaling | Cytokine Signaling |
| R-HSA-197264 | 0.003329 | Nicotinamide salvaging | Metabolism of vitamins and cofactors |
| R-HSA-168638 | 0.003714 | NOD1/2 signaling | Innate Immune Sys. |
| R-HSA-622312 | 0.004372 | Inflammasomes | Innate Immune Sys. |
| R-HSA-73621 | 0.004372 | Pyrimidine catabolism | Nucleotide metab. |
| R-HSA-8851680 | 0.01413 | Butyrophilin (BTN) family ints. | Adaptive Immune System |
| R-HSA-417957 | 0.02289 | P2Y receptors | Signaling by GPCR |
| R-HSA-168643 | 0.02381 | NLR signaling pathways | Innate Immune System |
| R-HSA-70171 | 0.02551 | Glycolysis | Metabolism of carbohydrates |
| R-HSA-5633008 | 0.02686 | TP53 reg. of cell death | RNA Polymerase II Transcription |
| R-HSA-416700 | 0.03182 | Other semaphorin ints. | Nervous system development |
| R-HSA-164952 | 0.03514 | The role of Nef in HIV-1 replication and disease pathogenesis | Infectious disease |
| R-HSA-5621481 | 0.04289 | C-type lectin receptors (CLRs) | Innate Immune System |

**Supplemental Table 5 – The number of times signature genes identified in Baghela et al. (2022) or Pena et al (2014) were identified in SeptiSearch gene sets.** The signatures are provided and their frequency (i.e., the number of times the gene appeared) in SeptiSearch gene sets. Genes in bold typeface were represented in ≥ 10 gene sets).

| **Signature** | **Signature Genes (Times identified in Complete SeptiSearch Database)** | **% found >10X** |
| --- | --- | --- |
| CR | **S100A12 (31), UPP1 (27), RETN (26), HK3 (24), S100A8 (24), DHRS9 (21), LILRA5 (21), CYP1B1 (20), FCER1G (20), S100A9 (20), PSTPIP2 (19), MCEMP1 (17), FPR1 (17), GK (17), FPR2 (16), CAMP (15), SERPINA1 (15), MXD1 (14), CD14 (13), CXCL10 (13), MARCO (13), MGST1 (13), RAB13 (12), VCAN (12), CDK5RAP2 (11), CPVL (11), CST3 (11), PTGR1 (11), HIST2H2AA3/H2AC18 (10)**, DDIT4 (9), FCER2 (9), MYADM (9), PLAUR (9), ALCAM (8), ALDH1A1 (8), CD300LF (8), HPSE (8), LY86 (8), PPBP (8), RNASE1 (8), CA12 (7), CD93 (7), HBEGF (7), HK2 (7), HTRA1 (7), PDLIM7 (7), PLD3 (7), TLR7 (7), TSPAN4 (7), LILRA3 (6), LIPA (6), MT1F (6), SLC16A10 (6), SLC7A11 (6), TMEM158 (6), TREM1 (6), ADAM15 (5), GPNMB (5), GPR137B (5), MT1X (5), NRIP3 (5), DPYSL3 (4), EGR2 (4), EMR1 (4), EMR3 (4), IL18BP (4), OLIG2 (4), PANX2 (4), RHBDD2 (4), S100A4 (4), ADAMDEC1 (3), ANKRD1 (3), CCL7 (3), CYP27B1 (3), FBP1 (3), HIST1H1C (3), HIST2H2AC (3), ITGB8 (3), MT1G (3), MT1M (3), PROCR (3), PTGES (3), TGM2 (3), C19orf59 (2), CCL19 (2), CCL22 (2), HSD11B1 (2), MT1H (2), NEFH (2), PAPLN (2), RARRES1 (2), CCL1 (1), CCL24 (1), CST6 (1), CTSK (1), IL3RA (1), KIAA1199 (1), MMP7 (1), NQO1 (1), SERPINB7 (1) | 27.30% |
| Organ Dysfunction /Severity | **HP (34), S100A12 (31), GPR84 (28), ANKRD22 (26), SAMSN1 (25), S100A8 (24), BMX (22), CLEC4D (22), G0S2 (21), CXCL8 (19), PSTPIP2 (19), RGL4 (18), ELANE (16), PCOLCE2 (15), GCH1 (14), PRTN3 (14), FFAR3 (13), GGH (13), SMPDL3A (13), ADGRE1 (12), RAB13 (12), SLC39A8 (12), CDKN3 (11), CLIC4 (11), TIFA (11), EPHB1 (10), SLCO4A1 (10)**, TTK (9), CCRL2 (8), IGF2BP3 (8), SLAMF7 (8), SPATC1 (8), TIGD3 (8), ITGB4 (7), RHAG (6), CACNA2D2 (5), GRAMD1C (5), SLC28A3 (5), ATP1B2 (4), CFAP45 (4), CNTNAP3 (4), MORN3 (4), PSAT1 (4), SDC2 (4), SERPINF1 (4), BAIAP3 (3), FAM83A (3), RELL1 (3), TNIP3 (3), DNAJB5 (1), DSP (1), TCTEX1D1 (1) | 51.90% |
| Mortality | **MMP8 (32), PLSCR1 (23), MS4A4A (22), DHRS9 (21), SORT1 (20), AGFG1 (17), PLAC8 (17), SLC51A (17), CCR1 (16), IFIT1 (16), OAS2 (16), HGF (15), TRIB1 (14), HIF1A (13), HIP1 (13), PAG1 (13), IL1RAP (11), CYP27A1 (10), FAR2 (10), PDE4D (10)**, PHACTR2 (9), PLXNA3 (8), RGL1 (8), SIGLEC1 (8), OSBP2 (7), PLEKHF1 (7), SDHAF3 (7), GPD2 (6), ACP5 (5), CSGALNACT1 (5), PIAS2 (5), SFXN1 (5), KCNH3 (4), FLNB (3), PTP4A1 (3), PEAK1 (2), RASGEF1B (2), TTN-AS1 (2) | 52.60% |
| NPS | **HP (34), IL1R2 (32), S100A12 (31), GPR84 (28), IL18R1 (28), MMP9 (28), FKBP5 (27), IRAK3 (27), UPP1 (27), ANXA3 (25), CD177 (25), GYG1 (25), SAMSN1 (25), IL18RAP (24), VNN1 (24), IL1RN (23), OLAH (23), PGD (23), ALOX5AP (22), ARG1 (22), BCL2A1 (22), BMX (22), CA4 (22), CLEC4D (22), GADD45A (22), PFKFB3 (22), TDRD9 (22), CST7 (21), G0S2 (21), GRB10 (21), LILRA5 (21), NAIP (21), SERPINB1 (21), SOCS3 (21), TLR5 (21), ALPL (20), CR1 (20), MGAM (20), S100A9 (20), ACSL1 (19), ETS2 (19), F5 (19), FCAR (19), NLRC4 (19), PFKFB2 (19), SLC11A1 (19), TSPO (19), DACH1 (18), FLOT1 (18), LRG1 (18), MCEMP1 (18), RGL4 (18), ZDHHC19 (18), AGFG1 (17), ASPH (17), CEBPB (17), DYSF (17), GALNT14 (17), IL4R (17), KCNE1 (17), NSUN7 (17), OPLAH (17), OSM (17), SLC51A (17), BASP1 (16), CD55 (16), FGF13 (16), FOLR3 (16), IDI1 (16), IER3 (16), IL1R1 (16), MKNK1 (16), NFKBIA (16), S100P (16), ST6GALNAC3 (16), ADGRG3 (15), ALOX5 (15), AP3B2 (15), AQP9 (15), ATP9A (15), EXOSC4 (15), FLOT2 (15), HGF (15), HPGD (15), ORM1 (15), PCOLCE2 (15), PDGFC (15), PPARG (15), SLPI (15), CD82 (14), CYP19A1 (14), CYSTM1 (14), DAAM2 (14), ECHDC3 (14), GCA (14), KCNMA1 (14), KREMEN1 (14), PGS1 (14), PROK2 (14), PYGL (14), TPST1 (14), ENTPD7 (13), FFAR3 (13), GAS7 (13), MYO10 (13), SIPA1L2 (13), BCL3 (12), DHRS13 (12), HSPA1A (12), KLHL2 (12), SULT1B1 (12), B3GNT5 (11), GRINA (11), IL1RAP (11), KL (11), MSRA (11), NECAB1 (11), PI3 (11), POR (11), PTGR1 (11), ROPN1L (11), SH3PXD2B (11), SLC1A3 (11), TP53I11 (11), CXCL1 (10), NIBAN1 (10), TRPM2 (10)**, ALDH1A2 (9), AMPH (9), CAPN13 (9), FUT7 (9), MANSC1 (9), MMP25 (9), ORM2 (9), PCSK9 (9), SYN2 (9), TMTC1 (9), TNFAIP8L3 (9), ZNF467 (9), ATP2C2 (8), CYYR1 (8), DGAT2 (8), GRAMD1A (8), IRAG1 (8), KAZN (8), NECTIN2 (8), PLB1 (8), SPATC1 (8), ANKRD55 (7), ARHGEF40 (7), CD163L1 (7), CYP1B1-AS1 (7), HPD (7), SEMA6B (7), WDFY3-AS2 (7), ADAMTS3 (6), CCNJL (6), FSTL4 (6), KLF14 (6), LYVE1 (6), PLIN5 (6), RFX2 (6), ST3GAL4 (6), TMEM120A (6), TMIGD3 (6), BTBD19 (5), HRH2 (5), INSC (5), LILRB5 (5), MTHFS (5), PLIN4 (5), ARMC12 (4), C3orf86 (4), CNTNAP3 (4), EPB41L4B (4), KCNE1B (4), LOXL1 (4), NEBL (4), PLK3 (4), ROM1 (4), SHROOM4 (4), SLC16A4 (4), SPP1 (4), XCR1 (4), DOK4 (3), LRRC70 (3), MAK (3), MLLT1 (3), NLRP6 (3), P4HA2 (3), SPINK8 (3), EFNA1 (2), FAM169B (2), IRAG1-AS1 (2), MIR646HG (2), NSMCE1-DT (2), PHF24 (2), AKR1C1 (1), BTNL8 (1), SPDYA (1) | 63.50% |
| INF | **MMP8 (32), ANKRD22 (26), OLFM4 (26), CEACAM8 (17), CRISP3 (16), DEFA4 (16), MS4A3 (16), PRTN3 (14), CXCL10 (13), ARL4A (12), P2RY14 (12), CD274 (11), NRN1 (11), ABCA13 (10), BNIP3L (10), MAOA (10), OLR1 (10), RGCC (10)**, ANKRD9 (9), CA1 (9), CHIT1 (9), IDO1 (9), MXI1 (9), AHSP (8), ALAS2 (8), ARG2 (8), C1orf116 (8), GMPR (8), PRDX2 (8), RBM38 (8), SNCA (8), ADD2 (7), ANK1 (7), CARD17 (7), FHL2 (7), GSPT1 (7), HSPH1 (7), OSBP2 (7), RIOK3 (7), SELENBP1 (7), SLC25A39 (7), SLC4A1 (7), TENT5C (7), TFEC (7), TSPAN5 (7), YOD1 (7), APOL4 (6), BPGM (6), CFH (6), DCAF12 (6), FBXO7 (6), FECH (6), FOXO3 (6), MKRN1 (6), RHAG (6), RNF11 (6), SIAH2 (6), SLC22A23 (6), SPTB (6), TFRC (6), TMEM40 (6), UBB (6), YBX3 (6), ACSL6 (5), ALDH5A1 (5), AOC1 (5), BCL2L1 (5), CR1L (5), CRISP2 (5), CTNNAL1 (5), EMID1 (5), EPB42 (5), GLRX5 (5), GYPB (5), HBM (5), HBQ1 (5), IFIT1B (5), ISCA1 (5), KANK2 (5), KRT1 (5), LGALS3 (5), MYL4 (5), NEDD4L (5), RAB3IL1 (5), RAP1GAP (5), RGS16 (5), SESN3 (5), SLC1A5 (5), SLC7A5 (5), STRADB (5), TMOD1 (5), UBE2O (5), XK (5), ADIPOR1 (4), ATP1B2 (4), BBOF1 (4), BLVRB (4), C8orf88 (4), CISD2 (4), ERFE (4), FAM104A (4), FHDC1 (4), GYPA (4), GYPE (4), HMGA2-AS1 (4), IGF2BP2 (4), KCNH2 (4), KLHDC8A (4), MRC2 (4), MROCKI (4), NFIX (4), NUDT4 (4), PGF (4), PNP (4), RNF175 (4), SEC14L4 (4), SLC14A1 (4), SLC2A1 (4), SLC6A8 (4), SMIM1 (4), SOX6 (4), TNS1 (4), TRIM58 (4), AQP1 (3), ARHGEF12 (3), ARHGEF37 (3), CA2 (3), CLIC2 (3), DMTN (3), DYRK3 (3), FAM210B (3), FAM83A (3), FAXDC2 (3), GPR146 (3), HEMGN (3), HEPACAM2 (3), ITLN1 (3), MARCHF8 (3), MFSD2B (3), MOSPD1 (3), PCDH1 (3), RAB6B (3), RFESD (3), RGS6 (3), RHD (3), RIPOR3 (3), SFRP2 (3), SMIM5 (3), SPTA1 (3), TBCEL (3), TGM2 (3), THEM5 (3), TIMP3 (3), TMC5 (3), TMCC2 (3), TRAK2 (3), USP12 (3), YPEL4 (3), ABCG2 (2), ACHE (2), BCAM (2), C9orf78 (2), CTSE (2), DNAJC6 (2), DPCD (2), FKBP1B (2), FRMD4A (2), GATA1 (2), HMBS (2), KDM7A-DT (2), KLC3 (2), KLF1 (2), LRRC2 (2), MBNL3 (2), OR2W3 (2), PBX1 (2), RUNDC3A (2), SELENOP (2), SGIP1 (2), SLFN14 (2), SMIM24 (2), TAL1 (2), TFR2 (2), TLCD4 (2), TLN2 (2), TRIM10 (2), TTC25 (2), ACKR1 (1), H2AW (1), HACD1 (1), KEL (1), PAGE2B (1), PLEK2 (1), PTPRF (1), RHCE (1), RNF182 (1), SLC6A9 (1), TMEM86B (1), TSPAN7 (1), TSPO2 (1) | 9.0% |
| IFN | **HLA-DMB (21), HLA-DPB1 (20), IFI44L (20), IFIT3 (20), OAS1 (20), XAF1 (19), HLA-DPA1 (18), CD74 (17), HLA-DMA (17), IFIT2 (17), MX1 (17), UBE2L6 (17), EPSTI1 (16), IFIT1 (16), IFITM3 (16), OAS2 (16), SERPING1 (16), STAT1 (16), GZMM (15), IFI35 (15), IFI6 (15), IRF7 (15), ISG15 (15), HLA-DRB1 (14), LY6E (14), OAS3 (14), BTN3A1 (13), DDX58 (13), HERC5 (13), HLA-DQA1 (13), LAP3 (13), MT2A (13), PLAAT4 (13), RSAD2 (13), RTP4 (13), TYMP (13), GBP4 (12), MYOF (12), LGALS3BP (11), OASL (11), PARP14 (11), PLEKHO1 (11), PSME2 (11), SPATS2L (11), WARS1 (11), ZBP1 (11), APOL1 (10), CMPK2 (10), HLA-DRB5 (10), HSH2D (10), OTOF (10), RNF213 (10)**, ABI3 (9), ADA2 (9), APOL3 (9), CIITA (9), EPHB2 (9), HAPLN3 (9), HES4 (9), IDO1 (9), JUP (9), PLD4 (9), ALDH1A1 (8), ARHGEF10L (8), BATF2 (8), CES1 (8), CSF1R (8), ETV7 (8), FRMD3 (8), KLHDC8B (8), PARP10 (8), PML (8), SEPTIN4 (8), SIGLEC1 (8), STAT2 (8), TCN2 (8), TIMM10 (8), USP18 (8), VAMP5 (8), CDKN1C (7), HERC6 (7), ISG20 (7), KLHDC7B (7), NUB1 (7), ODF3B (7), PARP12 (7), PNMA3 (7), SOCS1 (7), SP140 (7), TLR7 (7), TPPP3 (7), TRANK1 (7), ATF3 (6), CABP4 (6), CACNA1A (6), CYB561 (6), DHX58 (6), FANCA (6), GPBAR1 (6), LAMP3 (6), OSBPL5 (6), RGS12 (6), ST3GAL5 (6), TMEM150B (6), TNK1 (6), ZCCHC2 (6), ZNF618 (6), BLVRA (5), CALHM6 (5), FOLR2 (5), GALM (5), LGALS9 (5), PNPT1 (5), RUFY4 (5), S1PR2 (5), SHFL (5), SLC12A7 (5), STAC3 (5), TMEM176B (5), TMEM229B (5), ZNF239 (5), ZNF703 (5), AGRN (4), ASCL2 (4), CNDP2 (4), CYP4F22 (4), DDO (4), DNPEP (4), FZD2 (4), GRIN3A (4), MDK (4), MSR1 (4), NOD1 (4), SLC6A12 (4), SRC (4), AATBC (3), ATF5 (3), C2 (3), CBR1 (3), CBR3 (3), CMTR1 (3), CUL1 (3), EXOC3L1 (3), GRAMD1B (3), IL12RB2 (3), IL4I1 (3), KIF19 (3), MOV10 (3), RUBCN (3), SAMD4A (3), SCIMP (3), SDC3 (3), TMEM268 (3), TMEM51 (3), TTC21A (3), ZNF496 (3), APOBEC3F (2), ARHGAP22 (2), CDK18 (2), CKB (2), CLEC4F (2), EPS8L1 (2), LILRA4 (2), NPDC1 (2), P2RY6 (2), PCAT1 (2), RHEBL1 (2), TMEM255A (2), TMPRSS3 (2), UPK3A (2), WNT10A (2), XKR6 (2), AIFM3 (1), BISPR (1), CD40 (1), FIRRE (1), KLHDC7B-DT (1), OR52K1 (1), RTL5 (1), SLC27A3 (1), TGM1 (1), UBQLNL (1) | 28.60% |
| IHD | **GPR183 (19), FCER1A (18), CD24 (16), CCR3 (14), PTGDR (14), ID3 (13), MYBL1 (13), CD1C (12), FCMR (12), HLA-DOA (12), KLRF1 (12), AFF3 (11), CACNA2D3 (11), CPA3 (11), GOLGA8A (11), IL5RA (11), SGK1 (11), CLC (10), MS4A1 (10)**, ADAM28 (9), MEF2C (9), PTGDR2 (9), TBC1D9 (9), THEM4 (9), ZNF600 (9), AXIN2 (8), CACNA1I (8), CELSR2 (8), CRIP2 (8), HOPX (8), IL23A (8), MIR600HG (8), MYOM2 (8), PLXDC1 (8), PRSS33 (8), RPGRIP1 (8), TIGD3 (8), TPPP (8), ZNF395 (8), ADAMTS10 (7), CD200R1 (7), FAM169A (7), FBLN5 (7), FOSB (7), GOLGA8B (7), HPCAL4 (7), NR3C2 (7), PTPN13 (7), RECK (7), SLC29A1 (7), SMPD3 (7), SPRED1 (7), SRGAP3 (7), VSIG1 (7), ZNF540 (7), ZNF793 (7), ACACB (6), ADAM23 (6), AKT3 (6), CCDC65 (6), FBXL16 (6), GATA2 (6), GPR162 (6), GPR82 (6), HDAC9 (6), KLRC2 (6), MPP6 (6), SDK2 (6), SESN1 (6), SH3RF1 (6), SPNS3 (6), SPON1 (6), ZNF83 (6), ACVR2B (5), AGAP1 (5), ALOX15 (5), ANK3 (5), AZIN2 (5), C16orf74 (5), CACNG8 (5), CD19 (5), CNKSR2 (5), CYSLTR2 (5), DST (5), EBF4 (5), EEF2K (5), GOLGA6L9 (5), GRAMD1C (5), HRK (5), IGFBP3 (5), ITGA9 (5), KCNMB4 (5), MACROD2 (5), MTSS1 (5), NAP1L3 (5), NKX3-1 (5), PDZD4 (5), PI16 (5), PLEKHG4 (5), RAB40B (5), SAMD12 (5), STXBP4 (5), TNFRSF11A (5), WDR86-AS1 (5), WNT10B (5), ZNF10 (5), ZNF568 (5), ABCB4 (4), ACOT11 (4), AKAP12 (4), AMOT (4), C14orf132 (4), CCR4 (4), CD200 (4), CEP126 (4), CLCF1 (4), CMTM8 (4), CNR2 (4), COLGALT2 (4), CTSF (4), CYP4F12 (4), DEPTOR (4), KLHDC1 (4), MAP7 (4), MORN3 (4), NEFL (4), PLCB1 (4), PPFIA4 (4), SDC2 (4), SPEF2 (4), SPRY2 (4), TNFRSF21 (4), TPRG1 (4), UST (4), ZNF528 (4), ZNF85 (4), ACVR2A (3), ADGRD1 (3), AMOTL1 (3), ANGPT1 (3), ARL6 (3), CA8 (3), CACNB4 (3), CAMK2N1 (3), CLECL1 (3), DTX4 (3), DYNC2H1 (3), FGFR1 (3), GALNT12 (3), GATM (3), KLF8 (3), MATN1-AS1 (3), MYO6 (3), PLXNB1 (3), PODXL2 (3), PTPRM (3), SIGLEC8 (3), SLC29A2 (3), SOX13 (3), TMEM220 (3), TRIM2 (3), TTC30A (3), ZFYVE9 (3), ZNF382 (3), ZNF571 (3), ZNF577 (3), ZNF626 (3), ZNF823 (3), ANKRD18A (2), B4GALT6 (2), BAALC (2), BMPR1A (2), CCN3 (2), CLEC9A (2), CRIP3 (2), DSEL (2), GPR34 (2), KIT (2), NAP1L2 (2), NIPSNAP3B (2), PFN2 (2), PTGFRN (2), RNF207 (2), SELENOM (2), SPNS2 (2), TBC1D12 (2), ZFP37 (2), ZNF391 (2), ZNF525 (2), ZNF660 (2), ZNF665 (2), CAMSAP2 (1), CELSR1 (1), DIXDC1 (1), OPHN1 (1), PHEX (1), RAB44 (1), RHEX (1), SPATA6 (1), ZNF491 (1) | 9.50% |
| ADA | **CEP55 (16), TOP2A (16), JCHAIN (14), ASPM (13), CCNB2 (13), CD27 (13), GGH (13), TYMS (13), BIRC5 (12), HMMR (12), KIF11 (12), CCNA2 (11), CCNB1 (11), CDKN3 (11), CXCR6 (11), MKI67 (11), MZB1 (11), BUB1 (10), CXCR3 (10), DLGAP5 (10), OTOF (10), SHCBP1 (10), TK1 (10), ZWINT (10)**, ANLN (9), BUB1B (9), CDC20 (9), CDCA5 (9), CDK1 (9), CENPF (9), DUSP5 (9), RRM2 (9), SDC1 (9), TNFRSF17 (9), TPX2 (9), TTK (9), AURKA (8), CD38 (8), CDC25A (8), CDC45 (8), CDCA8 (8), ESPL1 (8), GTSE1 (8), ITM2C (8), KIF20A (8), MYBL2 (8), NEK2 (8), NUSAP1 (8), POLQ (8), SLC2A5 (8), TRIP13 (8), ALDH1L2 (7), CAV1 (7), CDCA3 (7), DEPDC1 (7), DTL (7), GINS2 (7), HJURP (7), HSP90B1 (7), KIF14 (7), KIF2C (7), MCM10 (7), MELK (7), NCAPG (7), NUF2 (7), PLAAT2 (7), PTTG1 (7), PYCR1 (7), SPC24 (7), UHRF1 (7), AURKB (6), CDC6 (6), CDCA2 (6), CDT1 (6), CENPM (6), CIT (6), CKAP2L (6), DEPDC1B (6), E2F7 (6), EXO1 (6), FAM3C (6), FOXM1 (6), GINS1 (6), GLDC (6), KCNN3 (6), KIF4A (6), KIFC1 (6), MCM2 (6), PDIA4 (6), PKMYT1 (6), SEL1L3 (6), SKA1 (6), SPC25 (6), TICRR (6), CDC25C (5), CENPA (5), CHEK1 (5), CHPF (5), CLSPN (5), COBLL1 (5), DIPK1A (5), EME1 (5), ESCO2 (5), HASPIN (5), IFI27L1 (5), KIF18B (5), KIF23 (5), KNL1 (5), LAG3 (5), MCM4 (5), NCAPH (5), OIP5 (5), ORC1 (5), RAD54L (5), SLC25A4 (5), SPAG5 (5), STMN1 (5), TPD52 (5), TROAP (5), TSHR (5), ZBTB32 (5), CCNE1 (4), CENPN (4), CTLA4 (4), DIAPH3 (4), E2F8 (4), EAF2 (4), GPRC5D (4), IFNG-AS1 (4), IGF1 (4), IGLL5 (4), KIF15 (4), LGMN (4), MANEA (4), MIXL1 (4), NET1 (4), PBK (4), PERP (4), PLK1 (4), POU2AF1 (4), SLC35F2 (4), STIL (4), TNFRSF13B (4), UCHL1 (4), ARHGAP44 (3), BRIP1 (3), CDCA7 (3), DERL3 (3), DSCC1 (3), FANCI (3), FEN1 (3), IRF4 (3), KLHL14 (3), MACC1 (3), MAD2L1 (3), MYO1D (3), PACSIN1 (3), PALD1 (3), PCLAF (3), PHGDH (3), PLK4 (3), SEC11C (3), TCF19 (3), UAP1 (3), UBE2T (3), ARHGAP11A (2), CENPU (2), CEP128 (2), DENND5B (2), ERCC6L (2), FAM30A (2), FBXO5 (2), HELLS (2), HID1 (2), NEIL3 (2), NUGGC (2), POLE2 (2), RAD51AP1 (2), SKA3 (2), SPATS2 (2), WDHD1 (2), ARHGAP42 (1), ARHGEF39 (1), BHLHE41 (1), CADM1 (1), CCNF (1), CHAC2 (1), CIP2A (1), CNKSR1 (1), COL4A4 (1), FAM111B (1), KIF18A (1), LMAN1 (1), ORC6 (1), PARM1 (1), PARPBP (1), POGLUT2 (1), SLC16A14 (1), SUV39H2 (1), TMEM200A (1) | 12% |
